# TEMPO in Solution, Melted, Solid, and Adsorbed on a Silica Surface: A Paramagnetic NMR Study

**DOI:** 10.1002/chem.202502979

**Published:** 2025-11-29

**Authors:** Ehsan Shakeri, Gabrielle E. Harmon‐Welch, Sara A. C. MacWade, Vladimir I. Bakhmutov, Janet Blümel

**Affiliations:** ^1^ Department of Chemistry Texas A&M University Texas USA

**Keywords:** melted TEMPO NMR, paramagnetic NMR spectra, solid TEMPO NMR, surface‐adsorbed TEMPO NMR, TEMPO

## Abstract

TEMPO (2,2,6,6‐tetramethyl‐1‐piperidinyloxy) is a stable radical that is phenomenologically interesting and of high importance in synthesis and catalysis. This radical has been studied extensively with EPR and used as a spin tag for biological materials and to probe hydrogen bonding. The NMR properties of TEMPO have not yet been reported. This contribution describes that all ^1^H and ^13^C NMR signals of TEMPO are visible in the paramagnetic NMR spectra. The ^1^H NMR spectra of TEMPO solutions have a chemical shift range of 50 ppm and require only 8 scans. The ^13^C NMR signals are localized within a chemical shift range of 2600 ppm and are obtained within 30 minutes. Samples prepared with different solvents and TEMPO concentrations have been investigated, and it has been demonstrated that all solvents undergo chemical shift changes due to the presence of TEMPO and adduct formation in the case of CDCl_3_. The impact of TEMPO on the ^31^P chemical shift of PPh_3_ is studied. Higher concentrations of TEMPO in solution lead to narrower signals and the molten substance, measured at 60°C, represents the culmination of this trend. It is demonstrated with paramagnetic solid‐state NMR that TEMPO adsorbs on a silica surface and displays the chemical shift and linewidth features of a dilute solution of TEMPO.

## Graphical abstract

Paramagnetic species can often be measured with EPR and NMR spectroscopy. This is demonstrated using the important radical TEMPO. All ^1^H and ^13^C NMR signals of TEMPO could be recorded and assigned. The impact of TEMPO on the chemical shifts of solvent signals and a phosphine is described.

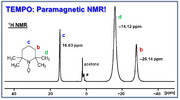

## 1 Introduction

**FIGURE 1:**
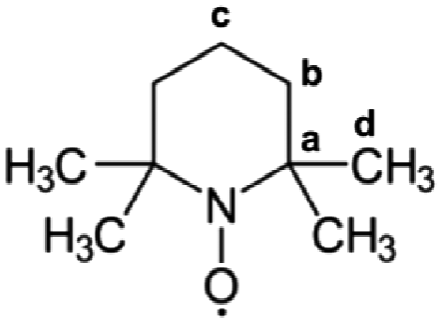
Structure of the stable radical TEMPO with an unpaired electron shared between nitrogen and oxygen in a two‐center three‐electron bond.

TEMPO (2,2,6,6‐tetramethyl‐1‐piperidinyloxy) (Figure 1) is a stable organic radical that is immensely important and widely applied in different fields. The applications and properties of TEMPO have recently been summarized in several reviews and dedicated publications [1, 2, 3]. They include the use as a catalyst for oxidation reactions, as a mediator for controlled radical polymerization, as a spin label in biological systems, as active materials in organic radical batteries, and as an antioxidant and free radical scavenger [1, 2, 3]. Furthermore, TEMPO has been employed to study hydrogen bonding effects with phenols [4]. Most recently, TEMPO and numerous derivatives thereof gained even more attention due to their application in Dynamic Nuclear Polarization (DNP), an NMR method which dramatically improves the signal‐to‐noise ratio of diamagnetic materials and compounds in solution [5, 6, 7, 8, 9].

Despite the importance of TEMPO and its many applications, the NMR spectra have not been reported in the literature. TEMPO is a textbook example for EPR spectroscopy of an organic radical [3, 9, 10], and therefore, it may be generally assumed that its NMR spectra can in principle not be recorded. However, there are numerous species whose electron relaxation time lies within the broad range that allows both EPR and NMR spectroscopy. This scenario has been described in general for transition metal complexes, for example, Cr(I) sandwich complexes in solution [11].

Since TEMPO features an unpaired electron (Figure 1), it requires paramagnetic NMR spectroscopy. The latter topic has recently been reviewed for paramagnetic transition metal complexes except metallocenes [12]. Our group and others have worked extensively with paramagnetic metallocenes in solution [13, 14, 15, 16, 17], in the polycrystalline form as neat metallocenes [18, 19] and solid solutions [17, 20], adsorbed on silica [21, 22, 23, 24], and embedded in polymers [25]. Hereby, besides solution NMR, solid‐state NMR spectroscopy [26, 27, 28, 29, 30] has been used as the most powerful analytical tool.

In contrast to metallocenes and other paramagnetic metal complexes, the paramagnetic NMR spectroscopy of organic radicals received much less attention [31, 32, 33, 34]. Early attempts to record paramagnetic NMR spectra often failed because the spectral window was not large enough. Too few scans were applied because it was not recognized that a very short pulse delay of 100‐200 ms can be chosen due to the short relaxation times of the paramagnetic species [17]. Additionally, while processing the spectra, phase correction, recognizing the difference between a background signal, a rolling baseline, and a broad paramagnetic signal can be challenging for nonexperts. For paramagnetic ^13^C NMR spectra of solids, in particular, proton decoupling and cross‐polarization (CP) should be avoided [18].

In this contribution, we show that the ^1^H and ^13^C NMR spectra of paramagnetic TEMPO can be obtained easily within minutes. No special equipment is required, and the NMR spectra were recorded using conventional high‐resolution NMR spectrometers and routine NMR programs and parameters. The ^1^H NMR signals are found within a comparatively narrow range and can be integrated due to the fast proton relaxation. We demonstrate that the spectrum calibration is best performed with a chemical shift standard in a commercially available capillary that prevents contact of the standard with the solution because of the impact of the paramagnetic species on the solvent signals. The overall ^13^C NMR chemical shift range of the TEMPO resonances is 2600 ppm, however, the signals of all carbon nuclei have been identified and assigned. Besides the solutions, melted, polycrystalline, and surface‐adsorbed TEMPO is investigated, and the signal halfwidths provide valuable information. The results presented in the following sections show that measuring TEMPO directly is straightforward and easy, and probably also possible during its many applications. Therefore, paramagnetic NMR provides an additional powerful analytical method for studying diverse systems containing TEMPO while using conventional NMR spectrometers.

## 2 Results and Discussion

### 2.1 Paramagnetic NMR Spectra of TEMPO in Solution

Regarding the importance and far‐ranging applications of TEMPO (2,2,6,6‐tetramethyl‐1‐piperidinyloxy), it is surprising that its complete NMR spectra have not been described yet. The compound is a textbook example for EPR spectroscopy and is used in graduate [10] and undergraduate teaching [3]. Adding the paramagnetic NMR spectra to the general knowledge base for organic radicals seems overdue.

**FIGURE 2:**
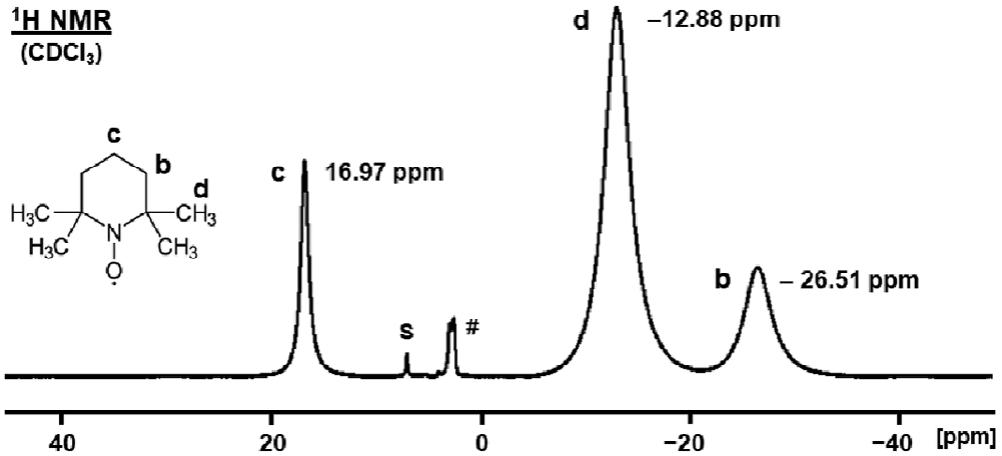
^1^H NMR spectrum of a dilute TEMPO solution in CDCl_3_. S denotes the solvent signal, # an impurity. The spectrum was calibrated using CDCl_3_ as an external standard.

The proton NMR spectrum of TEMPO can be obtained easily and one representative ^1^H NMR spectrum of TEMPO, dissolved in CDCl_3_, is displayed in Figure 2. The chemical shift and halfwidth data of ^1^H NMR signals obtained from different samples are summarized in Table 1. All ^1^H NMR spectra reported in this contribution were measured with only 8 scans using the standard single pulse program. No dummy scans were necessary to reduce the intensity of the solvent signals and the baseline could easily be corrected manually when necessary. Most importantly, the spectral window has to be much wider than for diamagnetic compounds. However, the number of data points should not be increased in order to avoid phase correction problems. Since the signals are broad, the digital resolution (Hz per point) will be sufficient.

**TABLE 1:** ^1^H NMR chemical shifts *δ*(^1^H) (ppm) and halfwidths **(∆ν**_1/2_) (Hz) of the signals **c**, **d**, and **b** of TEMPO in dilute and concentrated CDCl_3_, acetone‐*d*_6_, and toluene‐*d*_8_ solutions and of the melted and silica‐adsorbed substance. The chemical shifts were calibrated using the methyl protons of toluene as the standard in a sealed capillary that was centered in the 5 mm NMR tube (*δ*(^1^H) = 2.11 ppm). For calibrating the silica‐adsorbed TEMPO samples a trace of ferrocene was co‐adsorbed.

Sample	*δ*(^1^H) [ppm] (∆ν_1/2_ [Hz])		
	c	d	b
dilute CDCl_3_	16.97 (717)	‒12.88 (1868)	‒26.51 (2755)
dilute acetone‐*d*_6_	16.63 (470)	‒14.12 (1448)	‒26.14 (1810)
dilute toluene‐*d*_8_	15.67 (364)	‒13.96 (1279)	‒26.02 (1495)
conc. CDCl_3_	43.00 (219)	12.40 (466)	0.47 (304)
conc. acetone‐*d*_6_	39.79 (430)	9.09 (635)	‒2.69 (522)
melt (60 °C)	40.81 (252)	12.86 (311)	3.43 (168)
adsorbed on SiO_2_	16.51 (1215)	‒13.45 (1486)	‒27.84 (1306)

Since the signal‐to‐noise ratio was excellent (Figure 2) and the measurement required only 8 s with a pulse delay of 1 s, we did not strive to minimize the pulse repetition time, although the relaxation times of the TEMPO protons are much shorter. Due to the fast relaxation, the signal assignments for all ^1^H NMR signals are straightforward and can be based on the integrals, which are 2:12:4 from the downfield to the upfield signals. For the spectrum displayed in Figure 2, this corresponds to the signal for the protons in position **c** at 16.97 ppm, the methyl protons (**d**) at ‒12.88 ppm, and the methylene protons **b** at ‒26.51 ppm. This assignment is also corroborated by the fact that signals of protons with equal numbers of bonds separating them from the paramagnetic center are typically located on the same side of the diamagnetic region [14, 16]. The protons in positions **b** and **d** are three bonds removed from the location of the unpaired electron at the NO group, while the **c** protons have four bonds in between. It should be noted that the chemical shifts are comparatively close to the diamagnetic region and their halfwidths are moderate and not too different (Table 1). Therefore, the integrals are reliable because only minimal signal intensity is lost by the longer deadtime that a high‐resolution probehead requires as compared to a high‐power probe.

**FIGURE 3:**
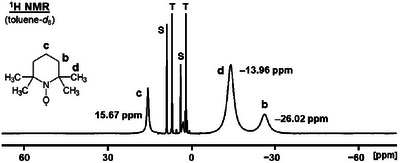
^1^H NMR spectrum of TEMPO dissolved in toluene‐*d*_8_ (**S**). **T** denotes the clipped proton signals of the chemical shift standard toluene in a sealed capillary that was centered in the NMR tube.

At this point, the calibration of the spectra needs to be addressed. The paramagnetic TEMPO leads to comparatively large chemical shift changes of the solvent signals [4, 35]. Therefore, the residual proton signals of the solvents cannot be used as internal chemical shift standards. In principle, it is possible to calibrate the NMR spectra by using an external chemical shift standard (Figure 2). Unfortunately, this requires one additional measurement, which is not time‐efficient [36]. Since the paramagnetic NMR signals are broad (Table 1), and moderate disturbances of the field homogeneity are not relevant, it is more convenient to use a solvent in a sealed capillary centered in the 5 mm NMR tube. The ^1^H NMR spectrum of TEMPO, dissolved in toluene‐*d*_8_, and calibrated with toluene in a capillary, is shown as a representative case (Figure 3).

**FIGURE 4:**
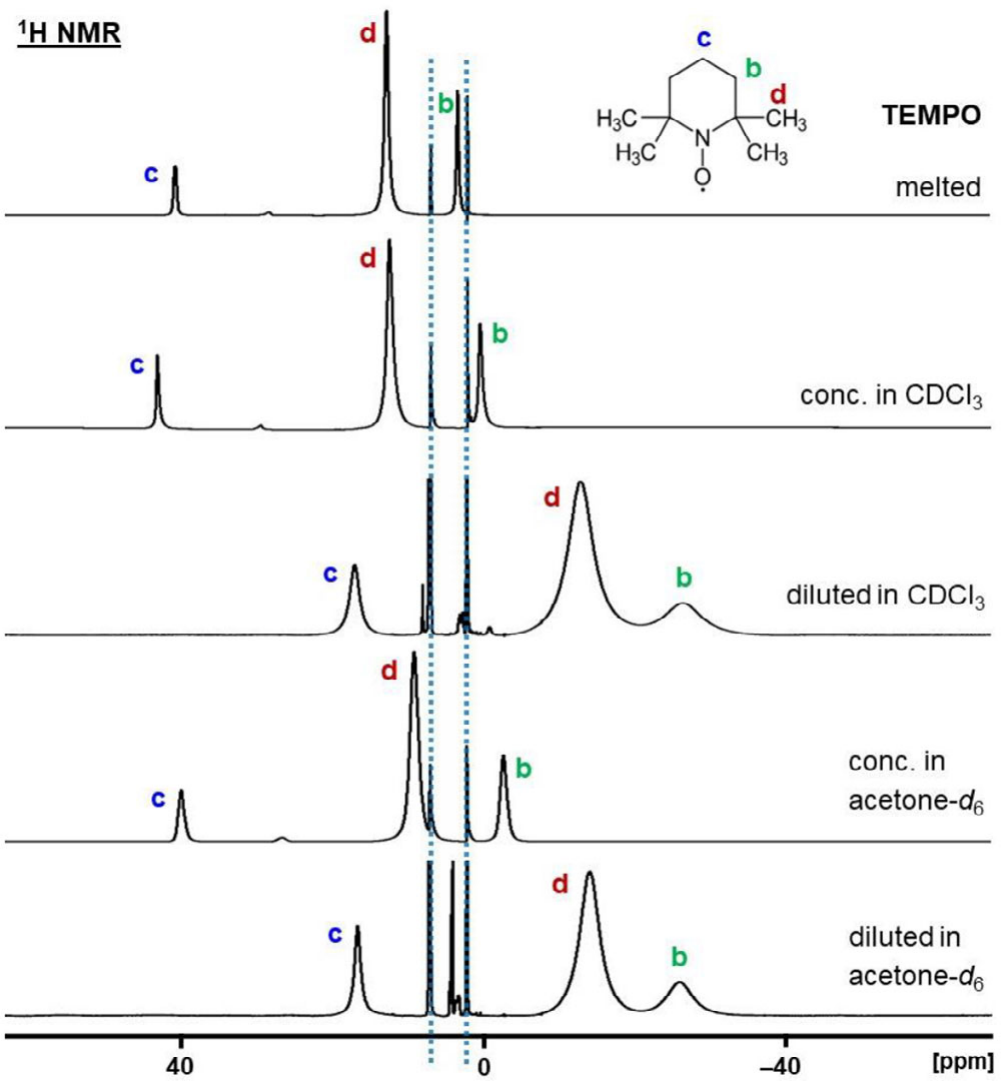
^1^H NMR spectra of melted TEMPO and TEMPO in dilute and concentrated CDCl_3_ and acetone‐*d*_6_ solutions. The dotted blue lines denote the signals of the chemical shift standard toluene in a capillary centered in the 5 mm NMR tube.

The ^1^H NMR spectrum displayed in Figure 3 illustrates several points. Most importantly, the chemical shifts of the solvent signals of toluene‐*d*_8_ (**S**) that interact directly with the paramagnetic TEMPO (8.90 ppm and 3.99 ppm) and toluene in the capillary (**T**) that is in the same space but not in direct physical contact with TEMPO (7.00 ppm and 2.11 ppm) differ by 1.90 ppm and 1.88 ppm for the aryl and alkyl protons. This is a substantial downfield shift in ^1^H NMR and confirms that the solvent signal cannot be used as an internal chemical shift standard, as it is common practice for diamagnetic samples. Therefore, toluene in a capillary centered in the NMR tube has been used for referencing all samples in the following (Table 1, Figure 4). Furthermore, the spectrum in Figure 3 shows that the impact of the capillary on the signal resolution is negligible. All toluene signals are very narrow as compared to the TEMPO signals and the halfwidths of the paramagnetic resonances do not change when the measurement is performed with a capillary. Finally, the spectrum in Figure 3 demonstrates that the chemical shift change of the solvent is not based on BMS (bulk magnetic susceptibility) effects [30] but stems from direct interactions of the paramagnetic TEMPO with the solvent molecules. One specific case of this solvent interaction will be discussed for CDCl_3_ below.

With the referencing of the spectra in place, next we studied the different factors influencing the chemical shifts and linewidths of the TEMPO signals. The impact of various solvents, concentrations, and temperature were surveyed. All ^1^H NMR signals of TEMPO measured under diverse conditions were obtained within the comparatively narrow range of 43 to ‒28 ppm (Figure 4, Table 1). Compared to other paramagnetic species, all signals are rather close to the diamagnetic region. For example, nickelocene displays a much larger ^1^H NMR chemical shift of ‒245.4 ppm [17, 25].

Interestingly, the nature of the solvent plays a minor role in the chemical shifts of TEMPO. As with diamagnetic solute/solvent pairs, the different susceptibilities of the solvents lead to slightly different chemical shifts of the TEMPO signals. When comparing the spectra of dilute solutions with similar concentrations of TEMPO in CDCl_3_, acetone‐*d*_6_, and toluene‐*d*_8_, the signals of the protons **c**, **d**, and **b** lie within the narrow ranges of 15.67 ppm to 16.97 ppm, ‒12.88 to –14.12 ppm, and ‒26.02 to –26.51 ppm (Figure 4, Table 1). Considering the overall chemical shift range of the paramagnetic signals, these solvent‐based differences of 1.3 ppm, 1.24 ppm, and 0.49 ppm, respectively, are very small. Furthermore, these chemical shift changes are far outweighed by the impact of the TEMPO concentration (Table 1), as discussed next.

In contrast to the minor solvent effects due to susceptibility differences of the solvents, increasing the concentration of TEMPO in the samples (Table 2) leads to substantial changes in the chemical shifts. All signals of the protons **c**, **d**, and **b** are now found downfield‐shifted to the regions 39.79 ppm to 43.00 ppm, 9.09 ppm to 12.40 ppm, and ‒2.69 ppm to 0.47 ppm (Figure 4, Table 1). Regarding, for example, the chemical shift changes of a dilute versus concentrated solution of TEMPO in CDCl_3_ reveal the massive differences in the *δ*(^1^H) for the proton signals **c** (26.03 ppm), **d** (25.28 ppm), and **b** (26.98 ppm) (Figure 4, Table 1). With increasing concentration, all ^1^H NMR signals experience a chemical shift change in one direction, downfield, and not just toward or away from the diamagnetic region. Interestingly, a noticeable, but much smaller, chemical shift change with the concentration had been described earlier for substituted cobaltocenes [15]. In that case, the chemical shift changes were different for each proton signal of the compound, and with increasing concentration all signals were shifted upfield [15]. For TEMPO, we find a downfield shift with increasing concentration, and all signals undergo similar chemical shift changes (Figure 4). The reason for the chemical shift changes is most likely the impact of the paramagnetic neighbors in more concentrated solutions that associate, albeit only temporarily, with a TEMPO molecule. Again, the solvent‐based chemical shift differences for the protons **c**, **d**, and **b** between the CDCl_3_ and acetone‐*d*_6_ samples are small with 3.21 ppm, 3.31 ppm, and 3.16 ppm. This corroborates the assumption that the impact of the concentration is dominant and the nature of the solvent plays a minor role.

**TABLE 2:** Amounts of TEMPO and solvents used for preparing the dilute and concentrated solutions.

Sample	TEMPO mg (mmol)	Solvent mg (mmol, mL)	TEMPO mg (mmol) per 1 mL of solvent
dilute CDCl_3_	30 (0.19)	1000 (8.4, 0.67)	45 (0.28)
dilute acetone‐*d*_6_	25 (0.16)	650 (11.0, 0.75)	33 (0.21)
dilute acetone‐*d*_6_ in rotor	58 (0.37)	197 (3.1, 0.23)	256 (1.6)
dilute toluene‐*d*_8_	40 (0.26)	600 (6.5, 0.64)	63 (0.41)
conc. CDCl_3_	400 (2.6)	50 (0.42, 0.033)	12000 (79)
conc. acetone‐*d*_6_	400 (2.6)	60 (1.0, 0.069)	5800 (38)

For paramagnetic signals, besides the chemical shifts, the signal halfwidths can provide valuable information about a sample. Often, the solvents play a role in this respect. In dilute solutions, the ^1^H NMR signals of TEMPO become narrower when changing the solvent from CDCl_3_ to acetone‐*d*_6_. CDCl_3_ leads to halfwidths of 717 Hz (**c**), 1.87 kHz (**d**), and 2.76 kHz (**b**) (Figure 4, Table 1). In acetone, the values are reduced to 470 Hz (**c**), 1.45 kHz (**d**), and 1.81 kHz (**b**). The reason for the smaller halfwidths in acetone‐*d*_6_ may be the lower viscosity of acetone (0.32 cP) as compared to chloroform (0.65 cP). The lower viscosity allows for a shorter correlation time of the molecules in solution and, consequently, reduced linewidths. Another reason for the narrower lines when using acetone as the solvent may be that CDCl_3_ can form aggregates with TEMPO, as the ^13^C NMR spectra discussed below indicate. This association with the solvent may slow down the molecular motion and lead to slightly broader lines. Overall, in analogy to the chemical shifts, the solvent effects on the linewidths pale in comparison to the impact of the concentration.

Next, we sought to identify the ^13^C NMR signals of TEMPO. Acetone‐*d*_6_ was chosen as the solvent because its diluted TEMPO solutions resulted in narrower ^1^H NMR signals than the CDCl_3_ solutions (Figure 4, Table 1). Furthermore, no complications due to additional solvent signals because of adduct formation, as observed with CDCl_3_ (see below), were expected for acetone‐*d*_6_ as the solvent. The ^13^C chemical shift range of paramagnetic species can be very large. For example, the ^13^C NMR signals of the cyclopentadienyl carbons of nickelocene derivatives are found in the range from 1400 to 1700 ppm [14, 16]. Similarly, the ^13^C NMR signals of nitronyl nitroxides are in the range from 1800 to 700 ppm [33, 34].

**FIGURE 5:**
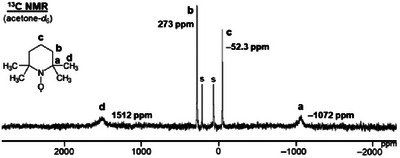
^13^C NMR spectrum of TEMPO in a dilute acetone‐*d*_6_ solution, recorded using a 4 mm MAS probehead without sample rotation. **S** denotes the clipped signals of the solvent. The probehead background signal has been subtracted.

Because of the expected large ^13^C NMR chemical shift range of TEMPO and the broad signals, care has to be taken when identifying the signals. In order to obtain reliable signal intensities and exclude complications by folding‐in resonances at the spectrum borders, the required large overall spectral range was obtained by measuring five individual segments with different irradiation frequencies under otherwise identical conditions and adding the spectra together. In principle, this can be performed at a high‐resolution NMR spectrometer. However, in order to reduce the number of required segments and therewith the measurement time, we used a solid‐state NMR instrument with high‐power capabilities to uniformly excite the ranges of the five individual spectra. The measurement time for each individual spectrum only amounted to 30 minutes because, in analogy to the ^1^H NMR measurements, the pulse repetition time could be short (200 ms). The liquid sample in a 4 mm rotor was not spun and no ^1^H decoupling was applied to avoid linebroadening effects [18] and allow signal assignments based on ^1^ *J*(^13^C‐^1^H) couplings. Figure 5 shows the obtained ^13^C NMR spectrum.

The ^13^C NMR spectrum displayed in Figure 5 features the desired four signals of TEMPO besides the solvent signals. Since no capillary could be applied to the 4 mm rotor, the spectrum was calibrated using C_6_D_6_ as an external chemical shift standard. The two resonances closest to the diamagnetic region at 273 ppm and –52.3 ppm have an intensity ratio of 2:1. Since no proton decoupling was applied that would distort the signal intensities due to the NOE effect, the halfwidths of the two signals are the same order of magnitude, and all paramagnetic resonances of TEMPO relax faster than the 200 ms relaxation delay, the intensity ratio can be used for the signal assignment. The resonance with lower intensity at –52.3 ppm resolves into a 1:2:1 triplet with a ^1^ *J*(^13^C‐^1^H) coupling of 106 Hz when no linebroadening is applied. Selective ^1^H decoupling with low power on the proton resonance **c** turns this resonance into a singlet. Furthermore, since carbon **c** (Figure 5) is furthest away from the unpaired electron, we assign this signal at –52.3 ppm, which is closest to the diamagnetic region, to carbon **c**. The resonance at 273 ppm can be assigned to the methylene carbon atoms **b** based on its intensity with respect to signal **c**.

The intensities of the two broad paramagnetic signals at 1512 ppm and –1072 ppm cannot be quantified in a straightforward manner, due to their large linewidths of 10 and 9 kHz, respectively (Figure 5). It is expected that some of the signal intensity, especially that of the broader resonance, is lost during the deadtime required by the probehead to avoid ringing effects. In general, the signals of carbons **a** and **d** should be on different sides of the diamagnetic region because of the different number of bonds that separate them from the unpaired electron [14, 16], and the opposite sign of the spin densities [16, 33]. We assign the signal at 1512 ppm to the carbons **d** and the signal at –1072 ppm to the quaternary carbons **a**. This assignment is based on the upfield shift of the signal of the quaternary carbon in nitronyl nitroxide radicals that resides next to the NO group and the downfield shift of the methyl carbon resonance [33].

Furthermore, the angle dependence of the coupling of the unpaired electron with carbons **d** and **b** over the covalent bond pathway can be used for the signal assignment [11]. The unpaired electron resides in a π orbital that is nearly perpendicular to the C**_a_**–C**_b_**–C**_b_**–C**_a_** plane of the TEMPO ring and the dihedral angle between the π orbital and the bond between carbons **a** and **b** is about 80° [37]. In analogy to the Karplus dihedral angle dependence of ^3^ *J* couplings in diamagnetic molecules, this angle is not favorable for the hyperfine coupling between the unpaired electron and carbon **b** [11]. Therefore, the ^13^C NMR signal of carbon **b** is only moderately downfield‐shifted to 273 ppm. On the other hand, the methyl carbons **d** feature a dihedral angle to the π orbital of about 36° with respect to the bond between carbons **a** and N [37]. This dihedral angle is more favorable for the coupling of the methyl carbons **d** with the unpaired electron and leads to a more pronounced downfield shift of their signals to 1512 ppm (Figure 5).

The halfwidths of the ^13^C NMR signals corroborate the tentative signal assignments (Figure 5). The large linewidth of 9 kHz belongs to the signal of the carbon nuclei **a**, which are closest to the unpaired electron. The resonance at 1512 ppm, corresponding to the carbons **d**, which are two bonds removed from the NO group, is 10 kHz broad. The methylene carbon signal **b** is much slimmer with a halfwidth of 480 Hz, due to the angle dependence of the coupling with the unpaired electron outlined above. The carbon nucleus **c** that is farthest away from the paramagnetic center is only 110 Hz broad.

### 2.2 Impact of TEMPO on Other Species in Solution

We already showed above that TEMPO changes the ^1^H NMR chemical shifts of solvents (Figure 3). Furthermore, increasing the concentration of TEMPO leads to substantial downfield shifts of its ^1^H NMR signals (Figure 4). In this section, we discuss the impact of TEMPO on the ^13^C NMR signal of the solvent CDCl_3_. Furthermore, for the ease of analysis, we will also use the ^31^P NMR resonance of PPh_3_ to probe the effect of the paramagnetic TEMPO on the chemical shift. It should be noted that chloroform is not a classical hydrogen‐bonding substance and PPh_3_ is not an electron acceptor.

**FIGURE 6:**
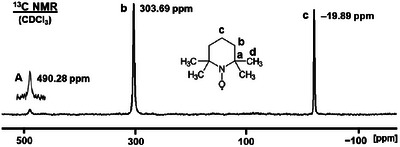
^13^C NMR spectrum of TEMPO (signals of carbons **b** and **c**) in a concentrated solution of CDCl_3_. **A** denotes the signal of a CDCl_3_ adduct of TEMPO.

First, the ^13^C NMR spectrum of a concentrated solution of TEMPO in CDCl_3_ was recorded, focusing on the spectral range around the signals of carbons **b** and **c** (Figure 6). Interestingly, a broad signal with a halfwidth of 576 Hz is visible in the spectrum at 490.3 ppm. This resonance only appears in spectra of chloroform solutions and none of the other solvents or the molten sample (see below) produces this signal. At the same time, the CDCl_3_ signal that would be visible at 77 ppm when measuring a diamagnetic sample is absent. Therefore, we assume that the signal at 490.3 ppm belongs to the TEMPO adduct of chloroform, with the hydrogen‐bonded structural element R_2_N‒O···D‒CCl_3_. A similar case has been observed earlier for chloroform hydrogen‐bonded to a phosphine oxide to form the structurally characterized adduct Cy_3_P═O···H‒CCl_3_ [38]. Obviously, the three chlorine substituents render the proton acidic enough to form an aggregate. A study by Shenderovich and Denisov showed that diverse substituted phenols associate with TEMPO, which leads to chemical shift changes of the aryl carbon signals [4]. However, the CDCl_3_ is only loosely and reversibly bound to the NO group of TEMPO. When the chloroform solution of TEMPO is diluted, the signal migrates upfield to 319.2 ppm. In summary, care has to be taken when recording paramagnetic NMR spectra in order not to mistake extremely shifted solvent signals for resonances that stem from the measured compound. In particular, solvents that allow for hydrogen bonding are not innocent.

**FIGURE 7:**
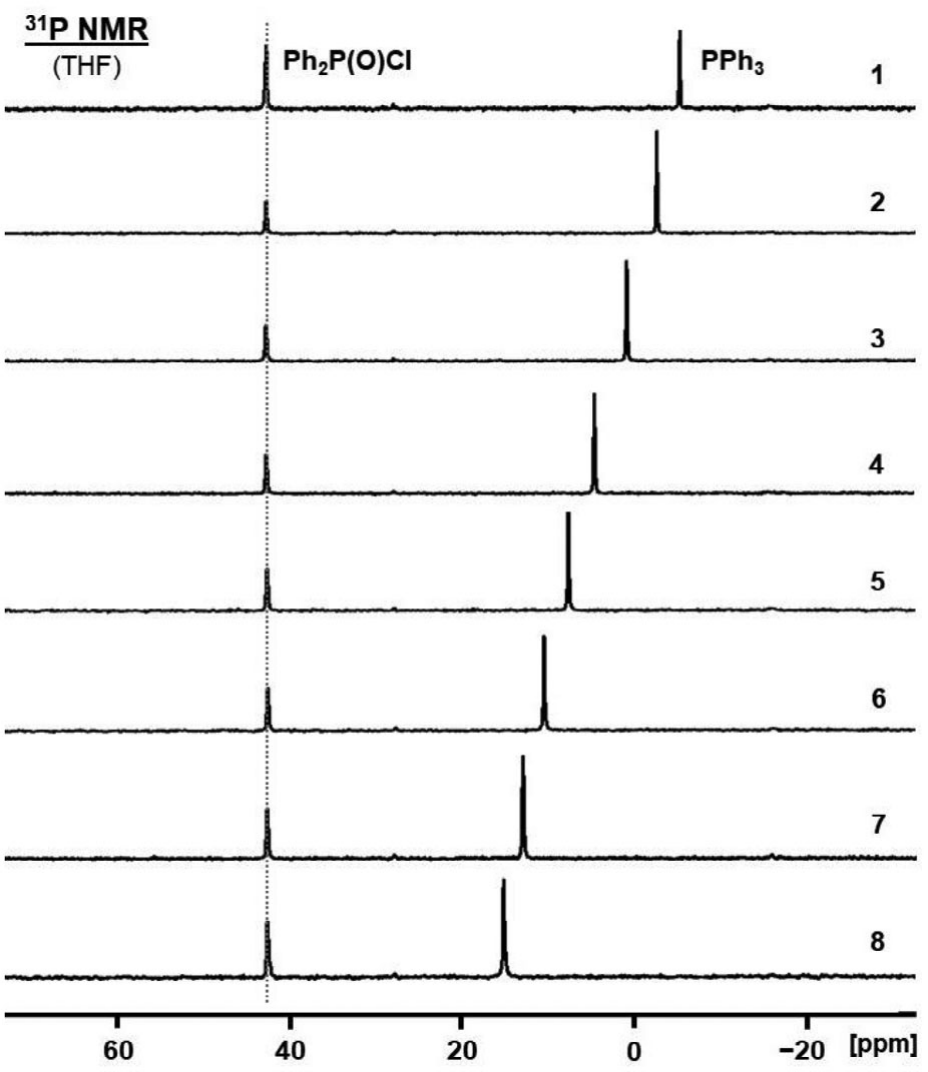
^31^P NMR spectrum of PPh_3_ in THF (top) and the spectra after adding aliquots of TEMPO (top to bottom). The trace of Ph_2_P(O)Cl in a capillary filled with Ph_2_PCl was used as the external standard.

Since TEMPO is used for signal enhancement in DNP, we also sought to probe its impact on the chemical shift of other species, for example, phosphines. The latter are important in coordination chemistry and as linkers for immobilizing catalysts [39, 40]. Importantly, TEMPO is a stable radical and does not react with PPh_3_. However, adding aliquots of TEMPO to a THF solution of PPh_3_ leads to an increase in the ^31^P NMR chemical shift value (Figure 7, Table 3).

**TABLE 3:** Amounts of TEMPO that were added to a solution of 10.3 mg (0.0393 mmol) PPh_3_ in 361.7 mg (0.407 mL) of THF (0.0966 mmol/mL).

Sample	TEMPO mg (mmol)	Molar Ratio TEMPO ∶ PPh_3_	*δ*(^31^P) (ppm)
**1**	0 (0)	0.0	−5.23
**2**	19.6 (0.13)	3.3	−2.55
**3**	46.8 (0.30)	7.6	−0.94
**4**	78.9 (0.50)	12.7	4.61
**5**	109.9 (0.70)	17.8	7.78
**6**	141.8 (0.91)	23.2	10.77
**7**	167.8 (1.07)	27.2	13.03
**8**	196.3 (1.26)	32.1	15.28

The chemical shift changes from –5.23 ppm to 15.28 ppm, more than 20 ppm, when a large amount of TEMPO is added. This is remarkable because paramagnetic metallocenes like nickelocene or cobaltocene only lead to negligible ^1^H and ^13^C NMR chemical shift changes of ferrocene, even when a ninefold excess of the paramagnetic species is applied [17]. Furthermore, PPh_3_ is not a strong electron acceptor and the interaction with the TEMPO radical is not obvious. It should be mentioned, however, that any oxidation of PPh_3_ can be excluded due to the gradual change of the chemical shift (Figure 7).

**FIGURE 8:**
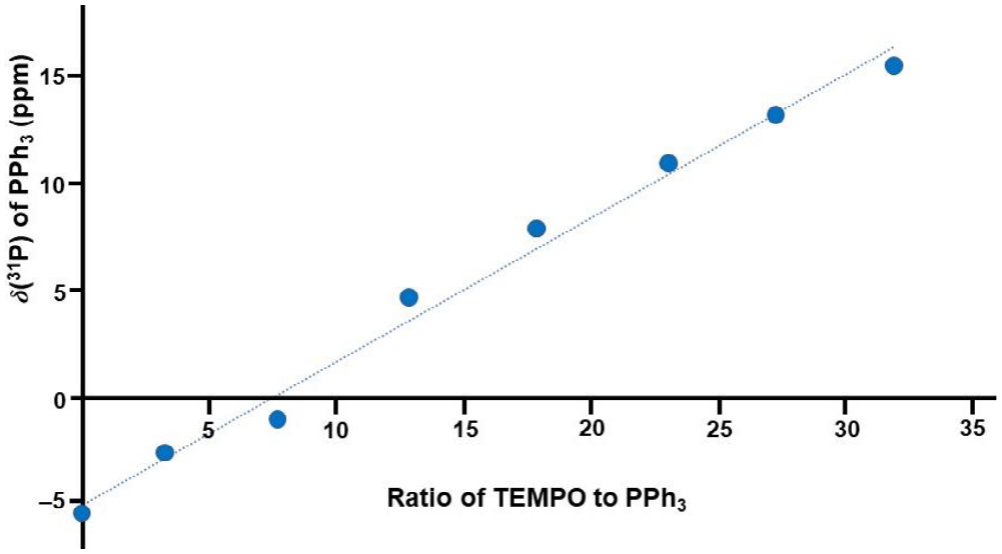
Linear correlation between the ^31^P NMR chemical shift of PPh_3_ (ppm) in THF and the amount of TEMPO added to the sample (Table 3).

Plotting the ^31^P NMR chemical shift values versus the concentration of TEMPO in the sample reveals a linear correlation (Figure 8). A linear correlation has been reported earlier on solvents co‐dissolved with paramagnetic cobaltocenes [15] and for phenols hydrogen‐bonding with TEMPO [4]. However, in these cases, the signals underwent upfield shifts [15], and were rather moderate [4], while the ^31^P NMR signal of PPh_3_ is shifted downfield substantially with increasing amounts of TEMPO (Figure 5, Figure 8, Table 3).

Interestingly, the PPh_3_ signal halfwidth is not increasing drastically when TEMPO is added. Overall, the signal linewidth only changes from 32 Hz to 42 Hz. This result indicates that TEMPO can also function as a potent, metal‐free, and inexpensive chemical shift reagent, being able to disentangle overlapping signals by drastic chemical shift changes, but not substantially broadening them.

### 2.3 Paramagnetic NMR Spectra of Melted TEMPO

**FIGURE 9:**
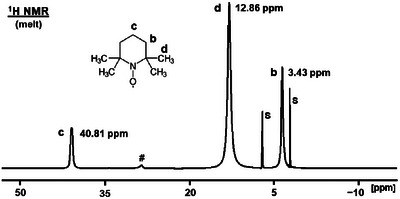
^1^H NMR spectrum of melted TEMPO recorded at 60°C. **S** denotes the proton signals of the chemical shift standard toluene in a sealed capillary that was centered in the NMR tube. # indicates an impurity.

**FIGURE 10:**
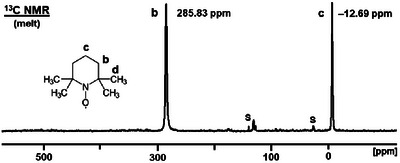
^13^C NMR spectrum of melted TEMPO (signals **b** and **c**) recorded at 60°C. **S** denotes the signals of the chemical shift standard toluene in a sealed capillary that was centered in the NMR tube.

We have described earlier that, in contrast to diamagnetic species, for paramagnetic metallocenes the signals get narrower when their concentrations are increased in solutions. Optimally, the neat compound is melted and measured as a liquid at elevated temperatures. This line‐narrowing effect on paramagnetic species has been demonstrated for diverse melted metallocenes [13]. Fortunately, the melting point of TEMPO is comparatively low (36°C to 38°C). Therefore, the substance could easily be melted and subjected to ^1^H and ^13^C NMR measurements at 60°C (Figure 9, Figure 10).

In order to calibrate the ^1^H and ^13^C NMR spectra in the absence of a solvent, we centered a sealed capillary containing neat toluene in the 5 mm NMR tube, as described above for solutions. Using this solvent as an external standard, but within the sample tube eliminates the problems arising from internal calibration, and no second measurement has to be performed with an external standard. The solvent toluene is favorable as the capillary standard for the melted substance because its signals do not overlap with those of TEMPO and it has a high boiling point of 110.6°C.

The ^1^H NMR spectrum of melted TEMPO shows the three signals for the protons **c**, **d**, and **b** at 40.81 ppm, 12.86 ppm, and 3.43 ppm (Figure 9). These values continue the trend of the *δ*(^1^H) with increasing concentration of the substance (Table 1). Hereby, the difference in the chemical shift values is smaller compared with those found for concentrated, than for dilute solutions. In other words, the melt represents the maximal concentration of TEMPO in the sample.

As expected, the higher temperature leads to the narrowest ^1^H NMR signals overall with 252 Hz (**c**), 311 Hz (**d**), and 168 Hz (**b**) (Table 1). Only the signal for **c** featured a slightly smaller halfwidth for the concentrated CDCl_3_ solution (219 Hz) than for the melt. From a practical perspective, recording the ^1^H NMR spectra of TEMPO in highly concentrated solutions is preferable because the slightly narrower lines hardly justify the additional effort required for heating up the probehead for the high‐temperature experiment.

In analogy to the proton NMR signals, the ^13^C NMR signals **b** and **c** of melted TEMPO are shifted toward the diamagnetic region due to the higher temperature (Figure 10) [41]. The ^13^C NMR signals of melted TEMPO appear at 285.83 ppm (**b**) and ‒12.69 ppm (**c**). The corresponding linewidths are 370 Hz (**b**) and 265 Hz (**c**).

### 2.4 Paramagnetic NMR Spectra of Solid TEMPO

**FIGURE 11:**
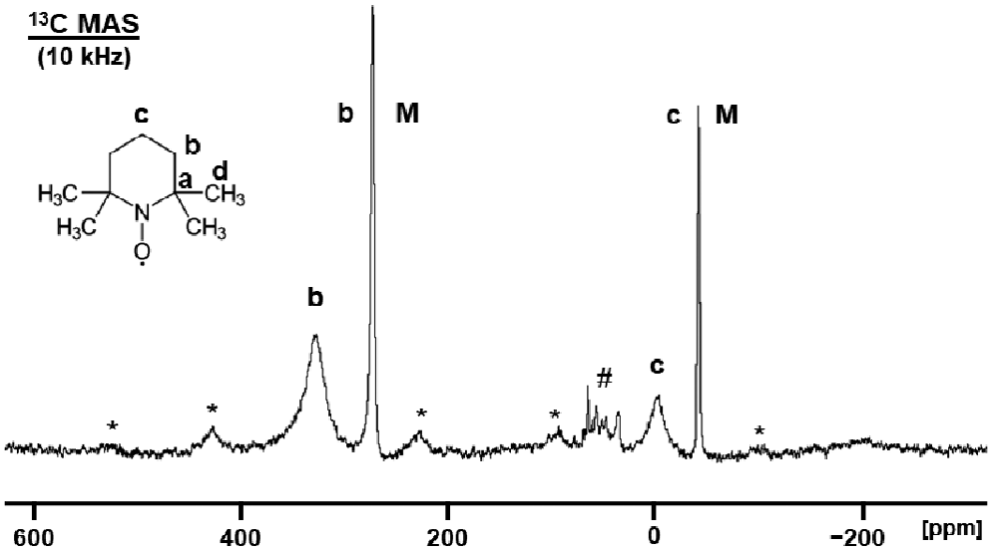
^13^C MAS NMR spectrum of polycrystalline TEMPO (carbons **b** and **c**) at 10 kHz spinning speed. The signals denoted with **M** represent the melted substance. # identifies signals of an impurity, the asterisks indicate rotational sidebands.

TEMPO has a low melting point of 36°C to 38°C. Therefore, care must be taken when rotating samples with high MAS (magic angle spinning) speeds. Due to the friction at the rotor wall, the sample will heat up about 20°C at a rotational speed of 10 kHz [18], bringing the substance into its melting range. Consequently, as a precaution, the TEMPO was measured in an HRMAS (high‐resolution magic angle spinning) rotor with tight inserts that are able to retain liquids [40, 42]. Figure 11 shows the ^13^C MAS spectrum of polycrystalline TEMPO, spun at 10 kHz. The narrow signals at 271.26 ppm and ‒43.27 ppm represent melted material. Due to the high mobility in the liquid, and the fact that higher concentrations lead to narrower signals, as demonstrated above, the linewidths are small with 369 Hz and 275 Hz, respectively. Furthermore, the signals of the melt do not feature any rotational sidebands, which means that the CSA (chemical shift anisotropy) [26, 28] was averaged out due to the rapid tumbling of the TEMPO molecules in the liquid domains. The signals of the melted substance increased in intensity with the measurement time and eventually were the only remaining resonances in the ^13^C MAS spectrum.

The ^13^C NMR signals **b** and **c** of the polycrystalline material are easy to identify in the spectrum (Figure 11). They are located at about 324.3 ppm (**b**) and ‒6.3 ppm (**c**). The residual linewidths of solid TEMPO are much larger than those of the signals in solutions or in the melt. The halfwidths are about 1607 Hz (**b**) and 1323 Hz (**c**). Both signals display first‐order rotational sidebands that have been identified by varying the rotational speed. The sidebands prove that the TEMPO remained solid at the beginning of the measurement because the CSA [26, 28] was not averaged out like in the melt or in solution. The first order rotational sidebands of signal **b** have about equal intensity. This symmetric signal shape is similar to that of the paramagnetic metallocenes nickelocene and chromocene [17, 18, 20].

Regarding the signals in Figure 11, it is interesting that the chemical shift differences between the signals of polycrystalline and melted TEMPO are substantial. For example, the resonance of the carbons **b** of polycrystalline versus melted material exhibits a chemical shift difference of 53.1 ppm. The signals **b** and **c** of melted TEMPO are not uniformly migrating toward the diamagnetic region. The methylene signal **c** is shifted upfield when TEMPO melts. Therefore, the chemical shift changes when transitioning from the crystalline to the melt domains cannot simply be explained in terms of the higher temperature in the melt. Rather, the environment of the molecules and the different phases may be responsible for the chemical shifts.

**FIGURE 12:**
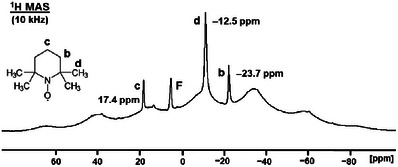
^1^H MAS spectrum of polycrystalline TEMPO. **F** denotes the trace of ferrocene that was added as an internal standard.

Next, we recorded the ^1^H MAS spectrum of polycrystalline TEMPO at 10 kHz rotational speed (Figure 12). Hereby, a trace of ferrocene‐*d*_2_ was added as an internal chemical shift standard. We made sure by ^2^H MAS NMR that the chemical shift of the ferrocene resonance was practically not changed by the presence of TEMPO. As the spectrum shows, large signals with their corresponding rotational sidebands are present that are broadened by the paramagnetic and homonuclear dipolar interactions. Due to the overlapping rotational sidebands and the presence of a background signal, only the signals of protons **c** at about 40 ppm and protons **b** at ca. –35 ppm can be identified unequivocally. On top of the broad signals, narrower peaks are visible at 17.4 ppm (**c**), –12.5 ppm (**d**), and –23.7 ppm (**b**) that correspond to the melted TEMPO and ferrocene (4.2 ppm) dissolved in it. The *δ*(^1^H) of these peaks is comparable to those of the signals of TEMPO in solutions (Table 1). The signal halfwidths are 233 Hz, 455 Hz, and 304 Hz for **c**, **d**, and **b**, respectively. These linewidths are similar to those obtained from concentrated solutions (Table 1).

### 2.5 Paramagnetic NMR Spectra of Surface‐adsorbed TEMPO

We have demonstrated earlier that diverse species can be adsorbed on silica, alumina, and carbon surfaces. This includes metallocenes [21, 22, 23, 24], alkanes [24], phosphines [43, 44], and phosphine oxides [45]. On the surfaces, these adsorbed species are very mobile and anisotropic interactions are averaged out by their reorientational motion on the curved surfaces within the pores. Therefore, the adsorbed molecules behave much like in solution and as a consequence the signals are often narrower than in the polycrystalline state. TEMPO has never been probed as a surface‐adsorbed species in a dry sample and the signals of adsorbed TEMPO have never been observed. Therefore, we explored whether this organic radical would be amenable to surface‐adsorption on silica.

**FIGURE 13:**
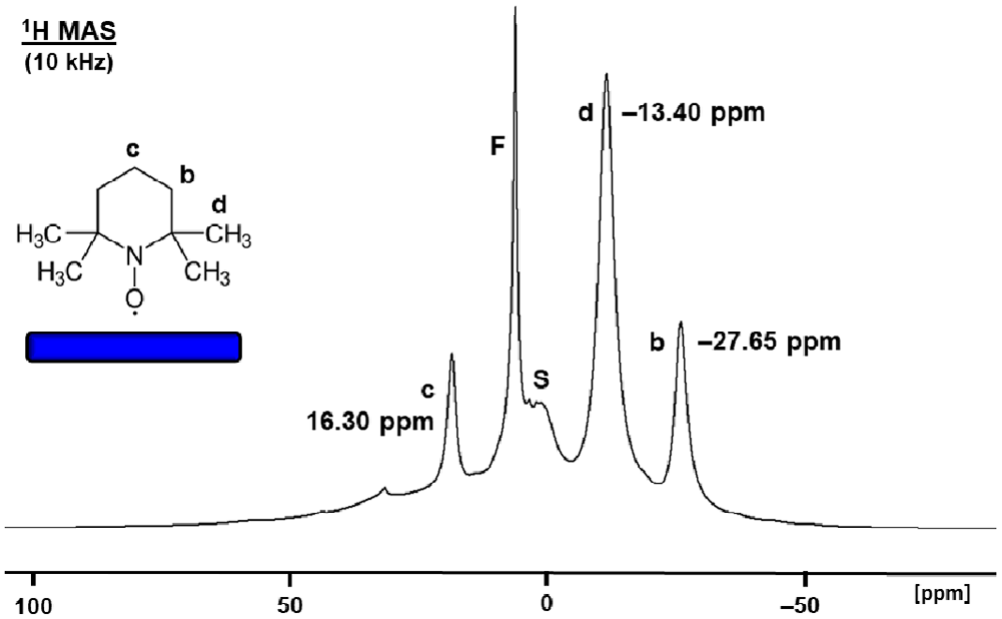
^1^H MAS NMR spectrum of TEMPO adsorbed on silica at 10 kHz spinning speed. The signal denoted with **F** corresponds to a trace of ferrocene added as an internal chemical shift standard. **S** denotes the ^1^H NMR signals of silica.

**FIGURE 14:**
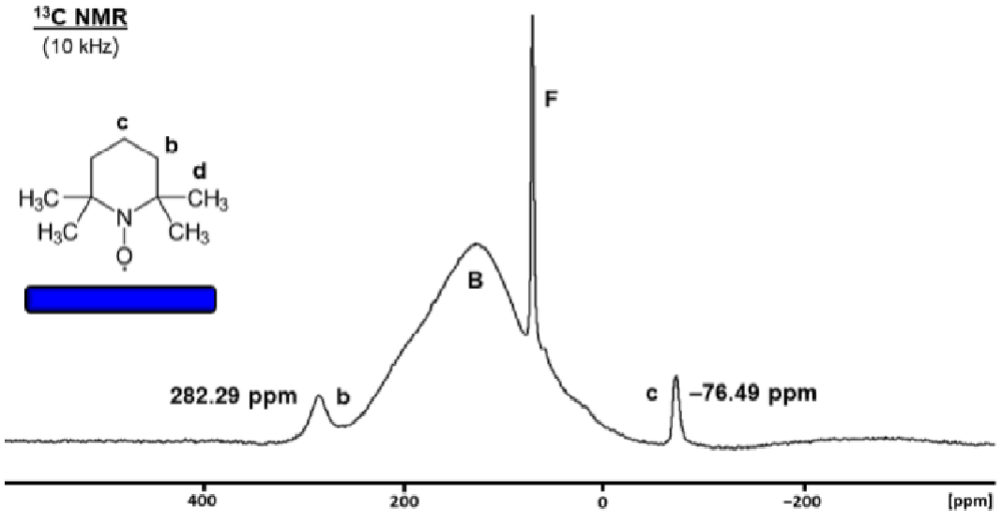
^13^C MAS NMR spectrum of TEMPO adsorbed on silica at 10 kHz spinning speed. The signal denoted with **F** corresponds to a trace of ferrocene added as the internal chemical shift standard. **B** denotes the background signal from the probehead, the Kel‐F cap and the rotor inserts.

Fortunately, as a stable radical, TEMPO does not decompose on the silica surface. In contrast to activated carbon [44], silica is diamagnetic and rather unreactive. TEMPO can be adsorbed on the surface from solution or by dry grinding of the components [21, 24] and subjected to solid‐state NMR analysis. As further advantages of adsorption, good signal resolution can be achieved and the melting problem of solid TEMPO is avoided when recording the MAS spectra of the adsorbed species. The ^1^H and ^13^C MAS spectra of TEMPO adsorbed with 40% surface coverage are displayed in Figures 13 and 14. Since we could not apply a capillary for the MAS measurement, the signals were calibrated with co‐adsorbed ferrocene as the internal chemical shift standard [20]. As mentioned above, ferrocene signals are practically not impacted by TEMPO. In contrast, the broad silanol proton signal of silica cannot be used as ^1^H chemical shift standard because of hydrogen bond formation with TEMPO and consequently a changed chemical shift [4]. In the ^13^C MAS spectrum, the background signal is too broad to serve as the chemical shift standard (Figure 14).

In analogy to the other adsorbable species mentioned above, TEMPO is mobile on the surface and anisotropic interactions are averaged out. None of the signals features rotational sidebands in the ^1^H MAS or ^13^C MAS NMR spectra.

The ^1^H MAS signals **c**, **d**, and **b** of adsorbed TEMPO are easily found at 16.30 ppm, –13.40 ppm, and –27.65 ppm, respectively (Figure 13). Comparing these *δ*(^1^H) of the signals of adsorbed TEMPO with the corresponding values of solutions and the melt, summarized in Table 1, it is obvious that they are very close to those of TEMPO in dilute solutions. This means that the silica surface mimics a solvent. Since the nature of the solvent does not have a major influence on the chemical shifts, the values are similar to those of chloroform and acetone solutions of TEMPO. Interestingly, the linewidth for the proton signal **c** (683 Hz) is comparable to those found in dilute solutions (364 Hz to 717 Hz). In contrast, the halfwidth of the ^1^H NMR signal of **d** (1.5 kHz) lies within the range for dilute solutions, while the resonance of **b** is even narrower (1.3 kHz). Due to its paramagnetism the TEMPO resonances do not overlap with the background signal of the silanol protons (Figure 13). Overall, surface adsorption can be chosen as a solvent‐free alternative for recording ^1^H NMR spectra of TEMPO at ambient temperature with solution‐type quality.

The ^13^C MAS spectrum of the signals **b** and **c** of surface‐adsorbed TEMPO is displayed in Figure 14. The TEMPO signal intensities are large compared to the background signal, which was not subtracted in this case. As in the ^1^H MAS spectrum, the signals of interest lie outside the diamagnetic and background signal region. The TEMPO signal at 282.3 ppm for carbons **b** corresponds well to the signal of the melted substance (285.8 ppm, Figure 10). However, the resonance for carbons **c** at –76.5 ppm appears upfield‐shifted by a record 63.8 ppm compared, for example, to the corresponding signal in the melt (–12.69 ppm, Figure 10). The signal shift for carbon **c** is closer to that obtained for a dilute acetone‐*d*_6_ solution (–52.3 ppm, Figure 5). As observed in the ^1^H MAS spectra, this speaks for the interpretation that surface‐adsorbed TEMPO resembles the dissolved species with silica acting as a solid solvent. It should be noted, however, that the mobility on the surface is not as high as that in solution. This is reflected in the larger halfwidths of the signals **b** (2.0 kHz) and **c** (734 Hz), as compared to the corresponding values in dilute acetone solution (480 Hz for **b** and 110 Hz for **c**, Figure 5).

## 3 Conclusion

In this contribution, we described for the first time how the paramagnetic ^1^H NMR and ^13^C NMR spectra of the important radical TEMPO can be obtained easily using a conventional solution NMR spectrometer. The time requirements for the ^13^C measurements equal those for diamagnetic compounds and the proton NMR spectra can be obtained within seconds. All ^1^H and ^13^C NMR signals of TEMPO have been identified and assigned.

The ^1^H chemical shifts and linewidths of TEMPO are dependent on the concentration. Higher concentrations lead to a downfield shift of all signals, while the linewidths decrease. Hereby, the nature of the solvent (chloroform, acetone, toluene) is less relevant. The spectrum of melted TEMPO resembles those of concentrated solutions.

Since the paramagnetic compound shifts the solvent signals downfield, a standard in a capillary centered in the NMR tube is recommended for calibration. In the case of chloroform, adduct formation with TEMPO leads to a downfield shift of several hundred ppm. Similarly, the ^31^P NMR resonance of PPh_3_ is shifted downfield by the presence of TEMPO and a linear correlation between the *δ*(^31^P) and the concentration of added radical is observed. TEMPO does not lead to oxidation of PPh_3_ in air, in contrast to the unpaired electrons on the surface of activated carbon [44].

The paramagnetic ^1^H and ^13^C MAS spectra of polycrystalline and surface‐adsorbed TEMPO on silica have been obtained. The chemical shifts and linewidths of the resonances of TEMPO on the silica surface resemble those of dilute solutions. This proves the high mobility of the molecules on the surface and suggests a future application as relaxation help for surface‐bound species like immobilized phosphines and catalysts [39, 40].

In summary, being able to record ^1^H and ^13^C NMR spectra of the paramagnetic TEMPO will allow it to be observed directly in different environments, not only by EPR, but also NMR. As we demonstrated earlier for nickelocene incorporated in an LDPE matrix [25], the paramagnetic signals of TEMPO should even be visible in low concentrations when incorporated, for example, in polymers, because they are far outside of the diamagnetic region. In future projects, we will also probe the application of TEMPO as a metal‐free, nontoxic, and inexpensive chemical shift reagent to disentangle overlapping polymer signals.

## 4 Experimental Section

### Sample preparation

TEMPO was purchased from Fisher Scientific and used as obtained. Since TEMPO is a stable radical, no inert gas atmosphere was required during sample preparation or storage. The amounts of solvents used for preparing the TEMPO solutions are summarized in Table 2.

For adsorbing TEMPO on a silica surface with 40% surface coverage, a solution of TEMPO (20.0 mg, 0.128 mmol) and ferrocene as an internal standard (23.7 mg, 0.127 mmol) in 2 mL of benzene was added to 304.6 mg of rigorously dried silica. After stirring for 30 min, the solvent was removed *in vacuo*. The samples with surface‐adsorbed TEMPO and ferrocene on silica were filled into a conventional 4 mm ZrO_2_ MAS rotor. The sample for the solid‐state NMR measurements of neat TEMPO was packed densely into an HRMAS rotor with tight‐fitting inserts.

### Solution NMR measurements

All solution NMR spectra were measured with conventional 400 MHz Bruker and 500 MHz Varian NMR spectrometers. For all ^1^H and ^13^C NMR measurements a standard single pulse sequence was used. The sweep widths were chosen large enough to include all signals and leave sufficient spectral range at the edges. The number of data points was kept as low as possible to facilitate phase and baseline corrections. The ^13^C NMR spectra were recorded without proton decoupling, unless mentioned otherwise. Usually, eight scans for ^1^H and 2000 scans for ^13^C with pulse delays of 1 s and 0.2 s, respectively, provided spectra with sufficient S/N ratios. The spectrum of melted TEMPO was obtained by pre‐heating and melting the substance in a 5 mm NMR tube and measuring at 60°C. All ^1^H and ^13^C solution and melt NMR spectra were calibrated by using toluene in a capillary that was centered in the 5 mm NMR tubes. For recording the complete ^13^C NMR spectrum, TEMPO was dissolved in acetone‐*d*_6_, filled into a 4 mm ZrO_2_ rotor with a tightly fitting cap, and measured at the instrument Bruker Avance Neo 400 in a MAS probehead without spinning and proton decoupling. Five spectral segments were measured separately and added together, as described in the text. The spectrum was referenced externally with C_6_D_6_. No lock or shim was used when recording any of the paramagnetic NMR spectra.

For studying the impact of TEMPO on the ^31^P NMR chemical shift of a phosphine, a sample containing 10.3 mg (0.0393 mmol; 0.0966 mmol/mL) of PPh_3_, dissolved in 361.7 mg (0.407 mL) of THF, was prepared in a 5 mm NMR tube. After the initial ^31^P NMR spectrum was recorded, aliquots of TEMPO with the amounts listed in Table 3 were added and the ^31^P NMR spectra were measured. As a chemical shift standard Ph_2_PCl (*δ*(^31^P) = 81.92 ppm) and traces of its oxide Ph_2_P(O)Cl (*δ*(^31^P) = 42.44 ppm) in a capillary centered in the NMR tube were applied. Each spectrum was acquired with 16 scans and a pulse repetition time of 1 s. No proton decoupling was applied and a single pulse program was used.

### Solid‐state NMR measurements

All ^1^H and ^13^C solid‐state NMR spectra were recorded on the spectrometer Bruker Avance Neo 400, equipped with a 4 mm MAS probehead and HRMAS rotors [42]. The ^13^C MAS spectra of neat and adsorbed TEMPO were recorded at 10 kHz spinning speed using a single pulse program without proton decoupling. A pulse delay of 0.2 s and 2000 scans yielded sufficient S/N ratios. The ^1^H MAS spectra were also recorded at rotational speeds of 6 and 8 kHz to distinguish the isotropic lines from rotational sidebands. Typically, 8 scans yielded satisfactory S/N ratios without applying linebroadening. For calibrating the ^1^H and ^13^C MAS spectra of neat and adsorbed TEMPO, silica‐adsorbed ferrocene‐*d*_2_ was used as the internal chemical shift standard (*δ*(^1^H) = 4.60 ppm, *δ*(^13^C) = 71.20 ppm) [23]).

## Conflicts of Interest

The authors declare no competing financial interests.

## Data Availability

The data that support the findings of this study are available from the corresponding author upon reasonable request.
